# Investigation of Atomic Level Patterns in Protein—Small Ligand Interactions

**DOI:** 10.1371/journal.pone.0004473

**Published:** 2009-02-16

**Authors:** Ke Chen, Lukasz Kurgan

**Affiliations:** Department of Electrical and Computer Engineering, University of Alberta, Edmonton, Alberta, Canada; University of Queensland, Australia

## Abstract

**Background:**

Shape complementarity and non-covalent interactions are believed to drive protein-ligand interaction. To date protein-protein, protein-DNA, and protein-RNA interactions were systematically investigated, which is in contrast to interactions with small ligands. We investigate the role of covalent and non-covalent bonds in protein-small ligand interactions using a comprehensive dataset of 2,320 complexes.

**Methodology and Principal Findings:**

We show that protein-ligand interactions are governed by different forces for different ligand types, i.e., protein-organic compound interactions are governed by hydrogen bonds, van der Waals contacts, and covalent bonds; protein-metal ion interactions are dominated by electrostatic force and coordination bonds; protein-anion interactions are established with electrostatic force, hydrogen bonds, and van der Waals contacts; and protein-inorganic cluster interactions are driven by coordination bonds. We extracted several frequently occurring atomic-level patterns concerning these interactions. For instance, 73% of investigated covalent bonds were summarized with just three patterns in which bonds are formed between thiol of Cys and carbon or sulfur atoms of ligands, and nitrogen of Lys and carbon of ligands. Similar patterns were found for the coordination bonds. Hydrogen bonds occur in 67% of protein-organic compound complexes and 66% of them are formed between NH- group of protein residues and oxygen atom of ligands. We quantify relative abundance of specific interaction types and discuss their characteristic features. The extracted protein-organic compound patterns are shown to complement and improve a geometric approach for prediction of binding sites.

**Conclusions and Significance:**

We show that for a given type (group) of ligands and type of the interaction force, majority of protein-ligand interactions are repetitive and could be summarized with several simple atomic-level patterns. We summarize and analyze 10 frequently occurring interaction patterns that cover 56% of all considered complexes and we show a practical application for the patterns that concerns interactions with organic compounds.

## Introduction

Protein-protein and protein-ligand docking are among the central topics in structural biology. The former provides useful input for constructing protein-protein interaction networks and for understanding the protein's function, while the latter provides a basis for selection of drug candidates by virtual screening [1], [2]. To date, interactions between proteins and macromolecules, i.e., protein-protein [3], [4], protein-DNA [5], and protein-RNA [6], have been systematically investigated. Thornton's study compared the size, shape, residue interface propensities and hydrophobicity of the protein-protein interface for four different types of protein-protein complexes [3]. Luscombe and colleagues studies the role of hydrogen bonds, van der Waals contacts and water mediated bonds in protein-DNA interaction. They concluded that the majority of the amino acid-base interactions observed follow general principles that apply across all protein-DNA complexes [5]. Rajamani and colleagues show that the anchor residues in protein-protein interactions maintain similar conformations before and after the binding, which allows for a relatively smooth binding process [7]. Ma's report shows that several structurally conserved residues could be used to distinguish between binding sites and general exposed surface; for instance, conservation of Trp, Phe, and Met residues on the protein surface was shown to be associated with a higher likelihood of formation of a binding site [8]. The principles that govern protein-metal ion interaction were recently reviewed by Dudev and Lim. They summarized several rules with respective to the coordination mode, coordination number, metal selectivity and coordination stereochemistry [9]. In another review by Dudev and Lim, various factors governing metal binding affinity and selectivity were systematically analyzed [10]. The structure and properties of the metal-binding sites were also discussed for specific metal ions like Ca^2+^ and Zn^2+^ [11], [12]. On the other hand, although characterization and prediction of protein-ligand interaction sites has attracted attention [13], [14], the protein-ligand interactions were never systematically studied and the rules that govern these interactions were not yet fully disclosed. The protein-ligand recognition is usually performed using an approach in which the protein and the ligand are considered as complementary surfaces [15], or by executing the actual docking process and calculating the protein-ligand interaction energies [16]. A recent study by Thornton's group reveals that pockets binding the same ligand show greater variation in their shapes than can be accounted for by the conformational variability of the ligand, which suggests that the geometrical complementarity is not sufficient to drive molecular recognition process [17]. This prompts our investigation into the interactions between proteins and ligands, in which we analyze both covalent bonds (normal covalent bonds and coordination bonds) and non-covalent bonds (electrostatic force, hydrogen bonds and van der Waals force). We focus on studying small ligands that exclude proteins, peptides, and nucleotides which were already investigated by other groups. Our aim is to find frequent regularities (patterns) that could be used to summarize interactions between the protein and the considered ligands. We analyze each of the major types of bonds for the ligands that are grouped into four categories including organic compounds, metal ions, inorganic anions, and inorganic clusters. In spite of inclusion of proteins characterized by low sequence identity and the diversity of the considered ligands, we found interesting and frequently occurring atomic-level patterns for several types of the considered ligands. We note that “atomic-level” term refers to the fact that patterns concern interactions between individual atoms of the residue and the ligand and it has no relation with the resolution of the considered crystal structures. Although the extracted patterns have been described in the literature, a comprehensive, in terms of the wide range of interaction and ligand types, overview of such interactions was not attempted. We systematically and conveniently summarize several major different interactions, we discuss specific details of these interactions across different residue types and ligands, e.g., the number of residues and the residues types that are involved in the coordination bonds with specific metal ions, and we quantify their relative abundance, which can be used to asses their importance in protein-ligand interactions. We also show, using a case study that concerns recent blind (without the knowledge of the ligand) geometric method for prediction of the binding sites, that usage of several patters in tandem improves the binding site predictions and that the sites predicted using patterns are complementary to the results based on the geometric analysis of the protein surface. Discovery of such interaction patterns would not only provide a comprehensive overview of protein-ligand interactions, but it would also facilitate design of binding site prediction methods and high-throughput molecular docking procedures.

## Materials and Methods

### Normal covalent and coordination bonds

The interaction between a non-hydrogen atom *A*_1_ of a residue and a non-hydrogen atom *A*_2_ of a ligand is defined as the covalent bond if the residue and the ligand do not have the opposite charge that would result in electrostatic force and the distance *d* of these two atoms satisfies
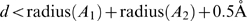
(1)where radius(*A_i_*) represents the radius of *A_i_*. As discussed by Davis and colleagues [18], in a typical 3Å resolution structure, the uncertainty of the position of the individual atoms can easily be 0.5Å or more. The marginal 0.5Å value used in formula 1 accommodates for the uncertainty of the positions of both atoms and for the variation of the length of covalent bonds, i.e., the length of a single bond between carbon atoms ranges between 1.2Å to 1.54Å.

Metal ions usually do not contain electrons in their outer shell. Therefore, if a metal ion forms the covalent bond with another atom, the pair of electrons shared by the metal ion and the second atom should be provided by the other atom. The corresponding covalent bond is defined as the coordination bond. As a result, metal ions and non-metal atoms (on a residue) whose interaction satisfies formula 1 are assumed to form the coordination bond.

### Hydrogen bond

Hydrogen bonds were calculated with HBPLUS [19]. To identify hydrogen bonds, the program finds all proximal donor (D) and acceptor (A) atom pairs that satisfy specified geometrical criteria for the formation of the bond. Theoretical hydrogen atom (H) positions of both protein and ligand are calculated with REDUCE program [20]. The criteria used for the current study are: H–A distance<2.7Å, D–A distance<3.5Å, D–H–A angle>90° and H–A–AA angle>90°, where AA is the atom attached to the acceptor.

### Electrostatic force

Among the 20 amino acids (AAs), the electrostatic force concerns positively charged Arg, His, and Lys residues and negatively charged Asp and Glu residues. The charge of the ligand is annotated using Protein Data Bank (PDB) [21] dictionary located at http://deposit.rcsb.org/public-component-erf.cif, which provides the charge of each atom of the ligand. An atom of the ligand and an AA in the protein are considered to exert electrostatic force with each other if they have opposite charges and at least one non-hydrogen atom of the AA is less than 3.5Å away from the charged atom of the ligand.

### Van der Waals force

A non-hydrogen atom *A*_1_ of a protein and a non-hydrogen atom *A*_2_ of a ligand form van der Waals contact if the distance *d* between these two atoms satisfies
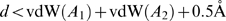
(2)where vdW(*A_i_*) is the van der Waals radius of *A_i_* and where these two atoms do not form covalent bond, coordination bond, hydrogen bond, and electrostatic force. This is consistent with the definition used in the investigation of protein-protein interactions by Ma and colleagues [8], in which two residues were considered to be in contact if there is at least one pair of atoms, one atom from each residue, at a distance smaller than the sum of their vdW radii plus a threshold of 0.5Å.

### The dataset of protein-ligand complexes and distribution of the ligands in PDB

The protein chains, which were selected using culledPDB list generated by PISCES server [22], are characterized by the following: 1) the chains share sequence identity of below 25%; 2) the resolution of the protein-ligand complex structure is below 2.0Å; and 3) the R_work_ value is below 0.25. These criteria, which resulted in selection of 2320 chains, assure that the selected proteins share low sequence identity (they adequately sample the sequence space) and that the corresponding structures have sufficient quality. The length of these chains varies between 20 and 1083, some short sequences are fragments of protein chains, and both monomers and oligomers are included. The protein and a ligand are assumed to interact with each other when at least one pair of non-hydrogen atoms, one from the protein and one from the ligand, can be found within 3.5Å distance. The minimal distance is consistent with the value used in [8]. If the same ligand binds a given protein in multiple pockets, all pocket-ligand complexes are included. Excluding the water molecule, all molecules annotated as “HET” in PDB, which includes organic compounds and ions, were taken as ligands. This excludes protein chains, peptides and nucleotides. As a result, 7759 pockets which have at least one contact with the considered ligand were extracted from the 2320 chains.

Among the 7759 complexes, some of the ligands appear multiple times, some are similar and could be grouped together and the same/similar ligands bind to a variety of pockets. To facilitate analysis of the protein-ligand interactions we select only these ligands that occur frequently and we group them into several categories. The ligands that bind to at least 100 pockets cover 59.4% of the considered complexes. Among these ligands, GOL, EDO, NAG, and ACT are organic compounds, Ca^2+^, Zn^2+^, Na^+^, Mg^2+^ and Cd^2+^ are metal ions, and SO_4_^2−^, PO_4_^3−^, Cl^−^, Br^−^ and I^−^ are inorganic anions. Additionally, some inorganic clusters, i.e., Fe-S cluster, also bind to a relatively large number of pockets. Therefore, the considered ligands (including those that occur in less than 100 pockets) are grouped into four categories: organic compounds, metal ions, inorganic anions, and inorganic clusters. We analyze total of 3685 organic compounds (that include 560 distinct types), 1682 metal ions (25 types), 1837 inorganic anions (19 types), and 54 inorganic clusters (9 types), which cover (3685+1682+1837+54)/7759 = 93.5% of all extracted pockets.

## Results

### Summary of the interaction patterns

**Figure 1:**
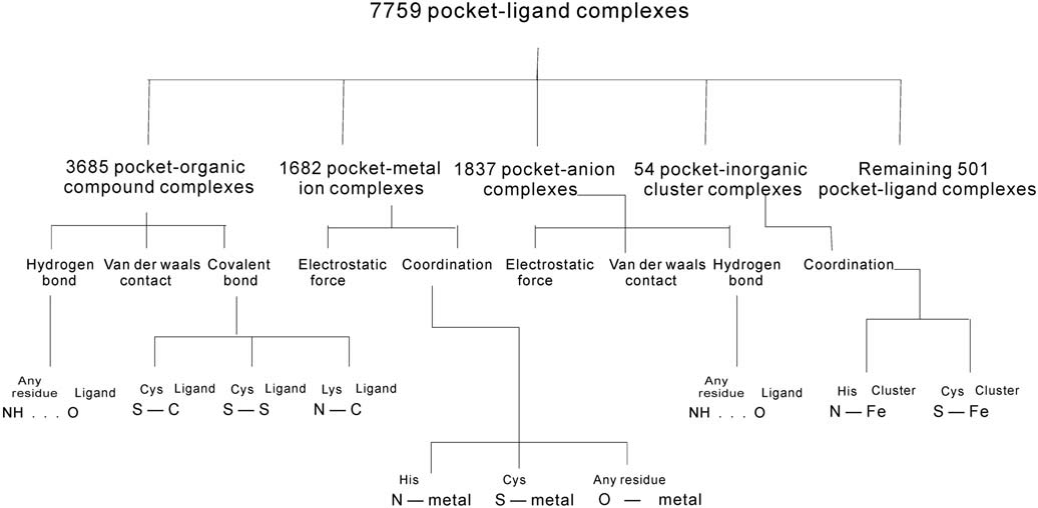
An overview of the protein pocket-ligand interactions. The top layer divides protein-ligand complexes into 5 major groups based on the type of the ligand. The second layer shows the major forces that are involved in formation of protein-ligand complexes for each type of the ligand. The bottom layer summarizes significant (frequently occurring) patterns for each force/bond type and each type of the ligand. The patterns are shown in X^R^…Y^L^ or X^R^ – Y^L^ format where X denotes an atom type of residue R in the protein, Y denotes an atom type of the ligand L, strong interactions (covalent and coordination bonds) are depicted by “–”, and weak interactions (hydrogen bond) are represented by “…”.

The protein pocket-ligand interactions are summarized in Figure 1. The top layer divides the 7759 protein pocket-ligand complexes into 5 categories based on the ligand type. The second layer lists the major forces that are involved in formation of protein-ligand complexes for a given ligand type. For instance, protein pocket-organic compound complexes are formed mainly by the means of covalent bonds, hydrogen bonds, and van der Waals contacts, which accommodate for 99.9% of the interactions. The remaining 0.1% of the contacts between a protein and the organic compound, which are omitted in the Figure 1, is based on the electrostatic force. The bottom layer provides significant patterns that are associated with interactions for a given type of the ligand and a given type of bond/force, which are discussed in detail in the following sections.

The forces that are omitted in Figure 1 are less significant (less frequent or nonexistent) for a given type of the protein-ligand interaction. Our analysis concentrates on the forces that are characterized by frequently occurring patterns for a given ligand category, while omitting some forces which are listed in Figure 1 and for which we could not find strong regularities (patterns). For the protein-organic compound interactions, we focus on the hydrogen and covalent bonds since they exhibit more regular and frequent patterns than the van der Waals contacts. In the case of the protein-metal ion interactions, electrostatic force and coordination bonds, which cover 95% these interactions, are analyzed. The discussion of the protein-inorganic anion interactions concentrates on the electrostatic force and hydrogen bonds; the van der Waals contacts are omitted due to lack of regular interaction patterns. Finally, our analysis of the protein-inorganic cluster interactions concerns only the coordination bonds since they constitute the main driving force for these interactions, i.e., they are involved in all considered protein-inorganic cluster complexes. Although we investigate all four interaction types, in our analysis we concentrate on the protein-organic compound and the protein-metal interactions since they occupy the largest fraction of the considered protein-ligand complexes and they are important for many biological processes [24], [25].

### Interaction patterns in protein-organic compound complexes

**Figure 2:**
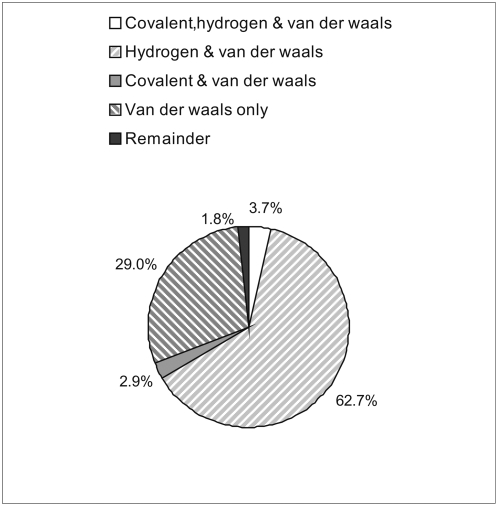
The summary of forces/bonds that are involved in formation of protein-organic compound complexes. The chart shows that most of the complexes involve multiple contact types with the most frequent contacts involving both van der Waals force and hydrogen bonds.

Organic compounds bind to proteins mainly by the means of the van der Waals contacts and the hydrogen bonds. Total of 85771 contacts were observed between an organic compound and a protein and they include 77554 van der Waals contacts, 7914 hydrogen bonds, and 246 covalent bonds. The remaining 0.1% of contacts are due to the electrostatic force. Among the 3685 protein pocket-organic compound complexes, 1067 complexes (29%) are based solely on the van der Waals contacts, 2309 (62.7%) involve both hydrogen bonds and van der Waals contacts, 107 (2.9%) incorporate covalent bonds and van der Waals contacts, and 135 (3.7%) include covalent bonds, hydrogen bonds, and van der Waals contacts, see Figure 2. We note that the number of hydrogen bonds is likely underestimated since REDUCE could not supply complete coordinates for hydrogen atoms of some ligands and thus some potential hydrogen bonds could not be counted.

#### Covalent bond

Majority of the 246 covalent bonds formed between organic compounds and proteins are summarized with four patterns: 1) 27 covalent contacts are formed between the thiol of Cys residue and the carbon atom of the organic compound (thioether bond); 2) 139 are formed between the nitrogen atom of Asn residue and the carbon atom of N-Acetyl-D-Glucosamine (NAG); 3) 28 concern the thiol of Cys residue and the sulfur atom of the organic compound (disulfide bond); and 4) 23 involve the nitrogen atom of Lys residue and the carbon atom of organic compound. We observe that the interaction between protein and NAG is established through the process of glycosylation and this interaction is not observed for other ligands. Therefore, this interaction is not included as a pattern for covalent bond. We denote the other three patterns as S^cys^—C^ligand^, S^cys^—S^ligand^, and N^lys^—C^ligand^ respectively. They cover (27+28+23)/107 = 73% of all investigated covalent bonds between proteins and organic compounds; see summary in Table 1. Both the thiol of Cys and the nitrogen atom of Lys could interact with a variety of organic compounds. This result indicates that the covalent bonds could be formed only between a few specific atoms of some AAs and a few specific atoms of the organic compounds.

**Table 1:** A summary of interaction patterns concerning covalent bonds formed between a protein and an organic compound.

Interaction pattern^1^	Average bond length (Å)	Occurrence	Ligands (organic compounds)
S^cys^—C^ligand^	1.83	27	3GC, 6NA, ACM, CYC, DBV, DKA, DPM, FAD, GOA, GVE, HC4, LBV, MKE, PEB, PLM, PYR, T10, XY2
S^cys^—S^ligand^	2.09	28	BME, DTT, SEO
N^lys^—C^ligand^	1.37	23	3PY, AZE, BGX, HPD, P3T, PBG, PLP, PYR, RET

1 The patterns are shown in X^R^ – Y^L^ format where X denotes an atom type of residue R in the protein and Y denotes an atom type of the ligand L.

Since some covalent bond patterns concern only a few dozens of complexes, we investigate whether they are specific to a certain protein family or more generic and associated with a variety of families. We note that in contrast to the covalent bonds, in the case of the subsequently discussed coordination and hydrogen bonds, thousands of contacts between the proteins belonging to a wide range of families and the ligands are established. Based on SCOP classification system [23], the S^cys^—C^ligand^ bonds are formed for proteins belonging to 15 families, which cover four major structural classes, i.e., all-α, all-β, α/β, and α+β. Similarly, the S^cys^—S^ligand^ and N^lys^—C^ligand^, bonds concern proteins from 15 and 10 families and 4 and 3 structural classes, respectively. This shows that the above patterns span dozens of structurally different protein families, which in turn indicates that they are not specific to a certain protein family or class.

Thioether bond and the bond between the nitrogen atom of Asn residue and the carbon atom of NAG are involved in a number of cellular activities and their formation could be associated with the protein's function. For instance, Ma's study suggests that the mycobacterium tuberculosis LipB enzyme transferase functions as a cysteine/lysine dyad acyltransferase, in which two invariant residues (Lys-142 and Cys-176) are likely to function as acid/base catalysts [24]. We observe that the tuberculosis LipB protein –decanoic acid complex is linked by thioether bond formed between Cys-176 and carbon-3 of decanoic acid. Zoltowski's study shows that formation of thioether bond between Cys thiol and the flavin C4a position is a response upon the blue-light excitation; attack of the thiol at C4a reduces the flavin ring, breaks aromaticity, and bleaches the absorption bands at 450 and 478 nm [25].. The above studies demonstrate the important role of the covalent bonds in catalysis, protein folding, and light-triggered cellular activity.

#### Hydrogen bond

Hydrogen bonds are formed in 2466/3685 = 66.9% of the organic compound based complexes. Although all 20 AAs can establish hydrogen bonds with compounds, their ability to form hydrogen bonds varies. Table 2 shows the distribution of occurrence of the hydrogen bonds formed by each AA and the occurrence of the AAs in the 3685 pockets. Seven hydrophilic residues (based on the low values of their hydropathy index [26]), including Arg, Lys, Asn, Thr, Ser, Gln, and His establish larger number of hydrogen bonds when compared their occurrence in the pockets. Moreover, six hydrophobic residues, i.e., Ala, Cys, Val, Ile, Met, and Leu, occupy 26.1% of the residues in the pockets and they form only 10.7% of the hydrogen bonds. This suggests that the hydrophilic residues form hydrogen bonds with the organic compounds more frequently when compared with the hydrophobic residues. Among the 7914 hydrogen bonds between proteins and organic compounds, AAs serve as donors for 6526 hydrogen bonds, and as acceptors for only 1371 hydrogen bonds; they serve as both donors and acceptors for the remaining bonds.

**Table 2:** A summary of hydrogen bonds formed between specific amino acids and organic compounds.

Amino acid	% hydrogen bonds with organic compounds	% of occurrence in binding sites	% hydrogen bonds with DNA molecules^2^	# hydrogen bonds		Hydropathy index value^1^
				as acceptor	as donor	
Arg	20.0%	7.5%	33.6%	29	1555	−4.5
Lys	10.4%	5.1%	14.8%	18	802	−3.9
Ser	8.8%	6.2%	10.1%	63	631	−0.8
Thr	8.0%	5.6%	8.2%	68	566	−0.7
Asn	7.6%	5.1%	7.9%	106	497	−3.5
Gly	6.8%	8.8%	3.7%	50	488	−0.4
Tyr	5.2%	5.9%	3.5%	69	346	−1.3
His	5.1%	4.0%	3.6%	91	312	−3.2
Asp	4.8%	5.7%	1.0%	278	103	−3.5
Gln	4.5%	3.3%	6.3%	75	282	−3.5
Glu	4.5%	4.9%	1.6%	300	53	−3.5
Ala	3.0%	5.6%	1.8%	38	200	1.8
Leu	2.2%	7.3%	0.4%	40	137	3.8
Trp	2.1%	3.6%	0.3%	13	156	−0.9
Val	2.1%	5.1%	0.7%	36	128	4.2
Ile	1.8%	4.4%	1.3%	27	113	4.5
Phe	1.2%	5.2%	0.4%	21	72	2.8
Met	0.9%	2.2%	0.4%	11	57	1.9
Cys	0.7%	1.5%	0.4%	9	43	2.5
Pro	0.4%	3.0%	0.1%	29	2	−1.6

1 the hydropathy index values from reference 26; the larger (smaller) the index values is, the more hydrophobic (hydrophilic) the amino acid.

2 the percentages of hydrogen bonds between specific amino acids and DNA molecules were taken from Table 2 of reference 5.

The distribution of occurrence of the hydrogen bonds with the organic compounds for the individual AAs is compared with the corresponding results obtained for protein-DNA interactions, which were derived based on 129 protein-DNA complexes [5], see Table 2. In both cases, the distributions are similar, i.e., Arg, Lys, Ser, Thr, and Asn establish the largest number of hydrogen bonds with both the organic compounds and the DNA molecules, while Phe, Met, Cys, and Pro establish the smallest number of hydrogen bonds with both types of ligands. The two AAs that establish the highest number of hydrogen bonds, Arg and Lys, are characterized by a larger relative number of bonds in the case of the binding with DNA, although we emphasize that the order of AAs in both cases is consistent. This suggests that the ability of AAs to establish hydrogen bonds could be an intrinsic characteristic of the AA itself, which is independent of the type of the ligand.

The negatively charged residues Asp and Glu did not exhibit strong affinity towards establishing hydrogen bonds in spite of having relatively high solvent accessibility and inclusion of two oxygen atoms in their side chains. We observe that Asp and Glu form the largest number of hydrogen bonds (278 and 300) when the AA serves as acceptor. At the same time they form only 103 and 53 hydrogen bonds when they serve as donors, which is relatively small when contrasted with the number of hydrogen bonds formed by other hydrophilic residues, e.g., 1555 for Arg and 802 for Lys. This low affinity to form hydrogen bonds could be explained by considering that the carboxyl groups of Asp and Glu often lend their H^+^ to solution, and as a result the two oxygen atoms on the carboxyl group are not bonded to hydrogen atom and cannot serve as donor when forming the hydrogen bond.

**Figure 3:**
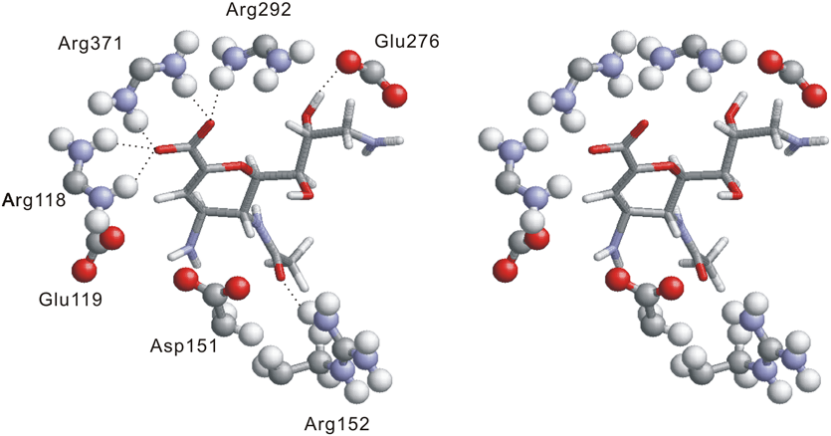
An example stereo diagram of hydrogen bonds formed between NH- group of a residue and oxygen atom of an organic compound. The oxygen atom is colored red, nitrogen atom is blue, carbon atom is gray, and hydrogen atom is white. The residues in the pocket are in ball and stick format while the ligand is in stick format. Hydrogen bonds are represented by “…”. The structure is taken from chain A of neuraminidase protein (PDB entry 1F8E), which interacts with 49A. The binding pocket contains four Arg residues and each residue contains 2 NH- groups. Three Arg residues (Arg118, Arg292, Arg371) are spatially adjacent, and they form five hydrogen bonds with the oxygen atoms of the ligand.

The most frequently formed hydrogen bond is established between NH- group (as the donor) of an AA and the oxygen atom of an organic compound. This type of the hydrogen bond covers 5206/7914 = 65.8% of all hydrogen bonds. To compare, the NH- group of organic compound serving as the donor and the oxygen atom of AAs account for only 325 hydrogen bonds. The surface patch that is characteristic for NH- group has high potential to form hydrogen bonds with organic compounds. For instance, in the chain A of neuraminidase protein (PDB entry 1F8E) [27], the pocket that binds 4,9-AMINO-2,4-DEOXY-2,3-DEHYDRO-N-ACETYL-NEURAMINIC (abbreviated to 49A in PDB) includes 4 Arg residues, i.e., Arg118, Arg152, Arg292, and Arg371, see Figure 3. Three of them, Arg118, Arg292, and Arg371, are spatially adjacent and they form 5 hydrogen bonds with the oxygen atoms of 49A, while the other residues in the pocket establish only 2 hydrogen bonds. The cluster of the five hydrogen bonds is crucial for the interaction between the protein chain and the compound.

#### Van der Waals contact

Majority of the van der Waals contacts are formed between carbon, oxygen and nitrogen atoms. These three atoms result in nine potential combinations which cover 94.8% of all van der Waals contacts between proteins and organic compounds. The most common van der Waals contacts are established between carbon atom of a residue and carbon atom of a compound, and carbon atom of a residue and oxygen atom of a compound. Each of the above two cases accounts for more than 25% of all van der Waals contacts. In contrast with the covalent and hydrogen bonds, van der Waals contacts are irregular and lack frequently occurring patterns that would indicate involvement of particular residues.

### Interaction patterns in protein-metal ion complexes

Among 1682 protein-metal ion complexes, 639 involve both coordination bonds and electrostatic force, 459 are based on electrostatic force but with no coordination bonds, and 499 incorporate coordination bonds with no electrostatic force. Overall, electrostatic force and coordination bonds are involved in (639+459+499) = 1597 complexes, which corresponds to 1597/1682 = 94.9% of all protein-metal ion complexes.

Asp and Glu residues are negatively charged and could potentially form electrostatic contact with the metal ions. Since the charge is not evenly distributed over the AAs, we analyzed which non-hydrogen atom of Asp/Glu is the closest to the metal ions. Among 1098 complexes involved the electrostatic force, metal ions formed electrostatic interaction with Asp and Glu 1511 times (in some complexes more than 1 electrostatic interaction is formed). In the case of 1385 out of above 1511 interactions, the oxygen atoms of the carboxyl group of Asp and Glu are the closest to the metal ion. This suggests that these two oxygen atoms could be more negatively charged than other atoms in the side chains.

Metal ions were observed to form coordination bonds with up to 6 atoms of a given protein, i.e., in chain A of 4-chlorobenzoyl coenzyme A dehalogenase protein (PDB entry 1NZY) [28], the calcium ion is coordinated with oxygen atoms of Gly49, Leu202, Ala203, Ala205, Thr207 and Gln210. On the other hand, some metal ions form coordination bonds with just one atom, i.e., in the chain A of human sex hormone-binding globulin protein (PDB entry 1D2S) [29], the calcium ion interacts only with His136. Total of 2345 coordination bonds are formed among the 1138 protein-metal ion complexes that involve this type of bond. The nitrogen atom in the side chain of His forms 787 bonds with the coordinating metal ions, sulfur atom of Cys forms 434 coordination bonds with metal ions, and oxygen atom (of any AA except Asp/Glu since interaction between metal ion and Asp/Glu is considered to be based on the electrostatic force) forms 1039 coordination bonds. The bonds based on these three atoms correspond to (787+434+1039)/2345 = 96.4% of all coordination bonds. The strong affinity of the oxygen to form coordination bonds with metal ion suggests that the interaction between the negatively charged Asp and Glu residues and metal ions could be a combination of both the coordination and the electrostatic force. The interaction between metal ions and Asp/Glu has been considered as coordination in many other studies. For instance, Angkawidjaja and colleagues reported that Ca^2+^ is coordinated by the side chains of Asp153, Asp157, and Gln120, and the carbonyl oxygens of Thr118 and Ser144 [30]; similarly, Declercq and coworkers show interaction between Ca^2+^ and the coordinating oxygen atoms of Asp51, Asp53, Ser55, Phe57, Glu59 and Glu62 [31]. As a result, the interactions between metal ions and Asp/Glu should be regarded as both coordination and electrostatic contacts if the distance between the corresponding atoms satisfies the definition of the coordination bond and the electrostatic contact.

Although the generic principles that govern protein-metal ion interactions were discussed in prior works [9]–[12], e.g., interactions concerning Cys-rich Zn^2+^-binding sites and affinity of interaction between Mg^2+^ and Asp/Glu in protein cavities [9], we could not find a systematic study that investigates how many residues and what residues types are involved (“preferred”) in the coordination bonds with specific metal ions, and that provides insights concerning similarities in the geometry of the coordination-based interactions with metal ions, which are discussed below.

Among the metal ions, Ag^+^, Ca^2+^, Cu^2+^(Cu^+^), Cd^2+^, Co^2+^(Co^+^), Fe^3+^(Fe^2+^), Hg^2+^, K^+^, Mg^2+^, Mn^2+^, Na^+^, Ni^2+^, Pb^2+^, Sm^2+^ and Zn^2+^ form coordination bonds with atoms of residues, see Table 3. Zn^2+^ forms coordination bonds in the largest number of pockets. This ion is coordinated by atoms of at most 4 residues in a given pocket and it favors to be coordinated by 3 or 4 residues. The second highest number of pockets that involve coordination bonds with a metal ion concerns Ca^2+^. These ions are coordinated by atoms of up to six residues in a pocket, and they prefer to form the coordination bonds with 4 or 5 residues. Coordination bonds with Mg^2+^ and Cd^2+^ ions involve 228 and 109 pockets, respectively. In contrast to Zn^2+^ and Ca^2+^, Mg^2+^ and Cd^2+^ ions form most of these bonds with atoms of 1 or 2 residues in a given pocket. Na^+^ ions form coordination bonds in 150 pockets and it favors to be coordinated by atoms of 3 or fewer residues. These 5 ions form coordination bonds in (426+328+228+109+150) = 1241 pockets, which constitutes 1241/1542 = 80.5% of all relevant pockets. The above results suggest that different metal ions prefer to be coordinated by a different number of residues in a given protein pocket.

**Table 3:** A summary of the coordination bonds between metal ions and a given number of residues in a protein pocket that contribute at least one atom to form the bond.

Metal ion	# pockets in which a given metal ion forms coordination bond with atoms of *x* residues						# of pockets for a given metal ion
	*x* = 6	*x* = 5	*x* = 4	*x* = 3	*x* = 2	*x* = 1	
Zn^2+^	0	0	120	123	74	109	426
Ca^2+^	24	70	84	44	50	56	328
Mg^2+^	1	0	14	59	73	81	228
Na^+^	1	5	17	44	41	42	150
Cd^2+^	0	1	5	7	26	70	109
Mn^2+^	0	1	16	24	20	24	85
Fe^3+^	1	4	15	28	6	5	59
K^+^	1	7	13	11	11	3	46
Cu^2+^	1	1	5	19	8	5	39
Ni^2+^	0	0	4	14	10	7	35
Co^2+^	0	0	4	9	5	0	18
Hg^2+^	0	0	1	0	6	9	16
Ag^+^	0	0	0	1	0	0	1
Sm^2+^	0	0	0	0	1	0	1
Pb^2+^	0	0	0	0	0	1	1

**Figure 4:**
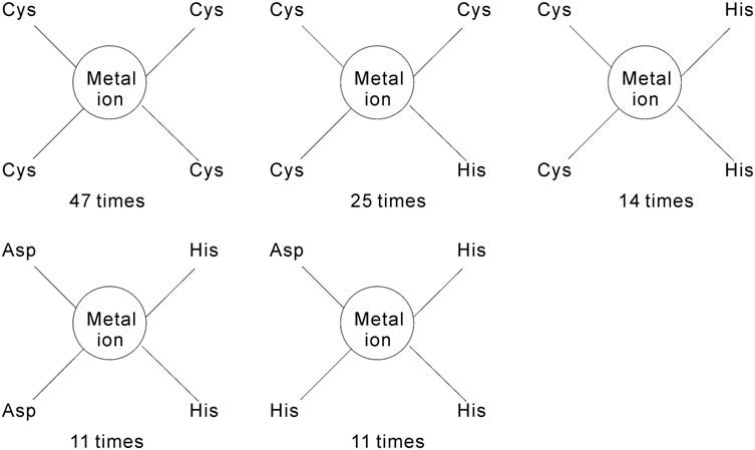
The residue groups that are coordinated by at least 10 metal ions and consist of 4 residues.

**Figure 5:**
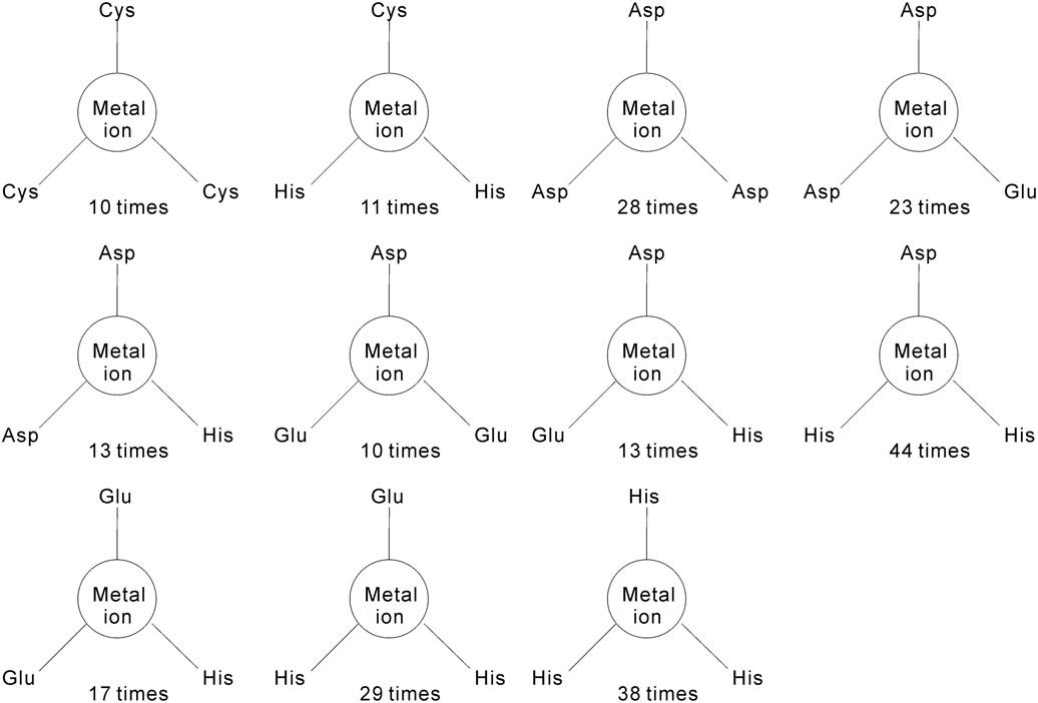
The residue groups that are coordinated by at least 10 metal ions and consist of 3 residues.

**Figure 6:**
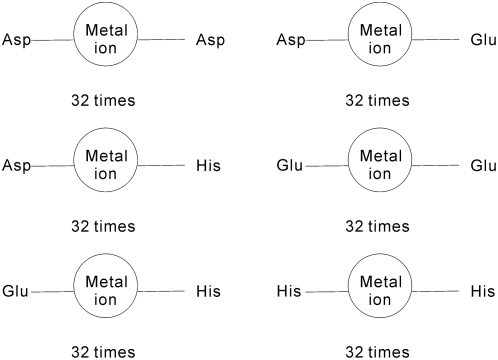
The residue groups that are coordinated by at least 10 metal ions and consist of 2 residues.

The residues which are coordinated by the same metal ion are grouped and we denote such groupings as the residue groups. We count the frequencies of the residue groups among different metal ions. For instance, given that Zn^2+^ forms coordination bonds with 4 Cys residues in 47 pockets, the corresponding frequency of (Cys)_4_ residue group is 47. The residue groups that are coordinated by at least 10 metal ions are shown in Figures 4, 5 and 6. The frequencies of residue groups that contain 5 or more residues are below 10 and thus they are not included in the above Figures. Total of 5 residue groups, i.e., (Cys)_4_, (Cys)_3_(His), (Cys)_2_(His)_2_, (Asp)_2_(His)_2_, and (Asp)(His)_3_, include 4 residues, see Figure 4. We observe that the (Cys)_4_ group is coordinated by the largest number of metal ions (47 metal ions). There are 11 residue groups that incorporate 3 residues, see Figure 5. These groups include (Cys)_3_, (Cys)_1_(His)_2_, (Asp)_3_, (Asp)_2_(Glu), (Asp)_2_(His), (Asp)(Glu)_2_, (Asp)(Glu)(His), (Asp)(His)_2_, (Glu)_2_(His), (Glu)(His)_2_ and (His)_3_. The (Asp)(His)_2_ and (His)_3_ groups are coordinated by the largest number of 44 and 38 metal ions, respectively. Finally, 6 residue groups, i.e., (Asp)_2_, (Asp)(Glu), (Asp)(His), (Glu)_2_, (Glu)(His) and (His)_2_, that make contact with 2 residues, see Figure 6.

**Figure 7:**
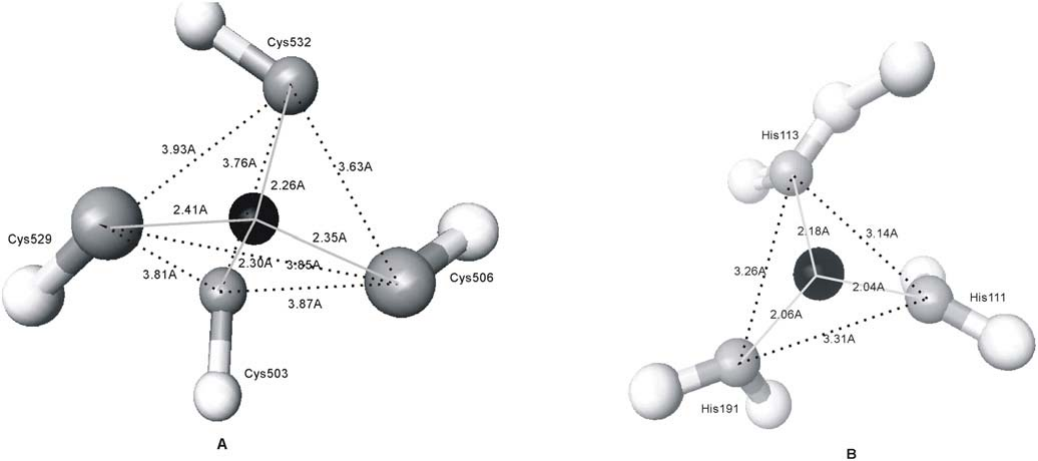
Examples of typical coordination bonds between metal ions and Cys and His residues. Coordination bonds are represented by solid lines; the dashed lines show the distance between atoms of different residues. Panel A shows the coordination bond between zinc ion and four Cys residues where sulfur atom is shown in gray, carbon atom in white, and zinc ion in black. The sulfur atoms of four Cys residues form an approximate regular tetrahedron and the zinc ion is located in its center. Panel B shows the coordination bond between zinc ion and three His residues. The nitrogen atoms are shown in gray, other atoms of the His side chain are in white, and zinc ion is colored black. The three nitrogen atoms form an approximate equilateral triangle with the length of the sides that varies between 3.14 Å and 3.31 Å. The zinc ion is not located on the triangle plane.

Cys and His are among the residues that the most frequently form coordination bonds with the metal ions. We observe that although the geometry of (Cys)_4_–metal ion and (His)_3_-metal ion interactions is different, each of these residue groups has similar geometry across the set of the corresponding pockets. The prevalent way to form the coordination bond between Cys and a metal ion involves four Cys residues arranged spatially close to each other to form a pocket; the metal ion is located in the center of this pocket. For example, in the chain A of PHD finger protein 21A (PDB entry 2PUY) [32], the zinc ion forms coordination bonds with Cys503, Cys506, Cys529, and Cys532. The distance between zinc ion and the sulfur atom of the four Cys residues varies between 2.26 Å and 2.41 Å. The four sulfur atoms form an approximate regular tetrahedron and the zinc ion is located in its center, see Figure 7A. The length of the tetrahedron edges varies between 3.63 Å and 3.93 Å. The coordination interaction between His and metal involves three His residues arranged to form a pocket with the metal ion located in approximately the same distance to the nitrogen atoms of these three residues. For example, in the chain A of Zn-dependent hydrolase protein (PDB entry 2R2D) [33], the zinc ion forms coordination bonds with nitrogen atoms of His111, His113, and His191. The distance between the zinc ion and the nitrogen atoms varies between 2.06 Å and 2.18 Å, see Figure 7B. The three nitrogen atoms form an approximate equilateral triangle with the length of the sides that varies between 3.14 Å and 3.31Å.

Metal ion-residue coordination plays a crucial role in stabilizing the protein structure and is involved in a number of catalytic activities [34]–[37]. Traoré's study reveals that the Zn(Cys)4 site locks the dimerization domain and stabilizes the dimer of protein PerR [34]. Ochiai and colleagues show that a calcium ion coordinated by Asp401, Glu422, His363, and His399 is required for the enzyme activity of rhamnogalacturonan lyase YesW [38]. Sankaranarayanan and colleagues point out that a zinc ion is directly involved in threonine recognition, forming a pentacoordinate intermediate with both the amino group and the side chain hydroxyl and mediated AA discrimination by threonyl-tRNA synthetase [39]. Covarrubias and coworkers demonstrate that depletion of a zinc ion, which is coordinated with aspartic acid side chain, leads to the lack of activity of Rv1284 gene [40]. The above example studies demonstrate the important role of metal ions in assisting protein folding and in catalysis of chemical reactions.

### Interaction patterns in protein-inorganic anion complexes

Inorganic anions bind to proteins mainly through electrostatic force, hydrogen bonds and van der Waals contacts. Among the 1837 anions, 1188 interact with the positively charged AAs such as Arg, His and Lys based on electrostatic interaction and 641 bind to the pocket by the means of hydrogen bonds and van der Waals contacts.

Similarly as in the case of metal ions, we studied which atoms of the positively charged residues are the closest to the inorganic anions. Among the 1188 protein-anion complexes that involve electrostatic force, 202 anions bind to His, 327 to Lys, and 659 to Arg. Nitrogen atom in the side chain of these three residues is the closest atom to the anion for 172 anion-His interactions, 222 anion-Lys interactions, and 565 anion-Arg interactions. These numbers suggest that the nitrogen atoms of positively charged residues may be closer to the center of the charge than other non-hydrogen atoms.

Among the anions that occur in PDB more than 100 times, 743 SO_4_^2−^ (743/948 = 78.5%) and 109 PO_4_^3−^ (109/148 = 73.6%) bind to positively charged residues, while some other anions less frequently bind with the charged residues. More specifically, 165 Cl^−^ (165/345 = 47.8%), 33 Br^−^ (33/126 = 26.2%), and 22 I^−^ (22/108 = 20.4%) bind to positively charged residue. This could be explained based on the formation of hydrogen bonds between the oxygen atoms of SO_4_^2−^ and PO_4_^3−^ and the NH- group of positively charge residues. For instance, PO_4_^3−^ forms 254 hydrogen bonds with positively charge residues (254/109 = 2.3 hydrogen bonds per pocket) and SO_4_^2−^ forms 1394 hydrogen bonds with positively charge residues (1394/743 = 1.9 hydrogen bonds per pocket). The combination of electrostatic force and hydrogen bonds stabilizes the anion-positively charged residue interaction.

Similarly to the protein-organic compound complexes, the most frequent hydrogen bond incorporates the NH- group of a residue that serves as the donor and the oxygen atom of a ligand. This pattern concerns 2777 hydrogen bonds which converts into 2777/3190 = 87.1% of all hydrogen bonds between a protein pocket and an inorganic anion.

### Interaction patterns in protein-inorganic cluster complexes

Amidst the nine types of inorganic cluster that could be found in PDB, FS4, FES, SF4, F3S, CLF, and FS3 are Fe-S clusters and contain only iron and sulfur atoms. The remaining three clusters, which include CFN, FSO, and NFS, also mainly contain iron and sulfur atoms.

We observe that coordination bonds are involved in all 54 protein-inorganic cluster complexes. These bonds are usually formed between the iron atom of the cluster and the sulfur atom of Cys residue, and the iron atom of the cluster and the nitrogen atom in the side chain of His residue. These two coordination bond patterns cover 201/204 = 98.5% of all coordination bonds between inorganic cluster and a protein pocket. Although FS4, SF4, F3S, and FS3 are positively charged and FSO is negatively charged, these clusters do not interact with charged residues. We did not find the electrostatic force based interactions between the inorganic clusters and proteins.

### Comparison between protein-protein interaction interfaces and protein-organic compound binding pockets

Several statistical studies have investigated the AA frequencies and the pairing preference for the protein-protein interaction interface. Ben-Tal's study indicates the hydrophobic residues are abundant in large interfaces while polar residues are more abundant in small interfaces [41]. They also conclude that contacts between pairs of hydrophobic and polar residues are unfavorable and that the charged residues tend to pair subject to charge complementarity. This conclusion was confirmed in a more recent study by Helms and colleagues [42].

**Figure 8:**
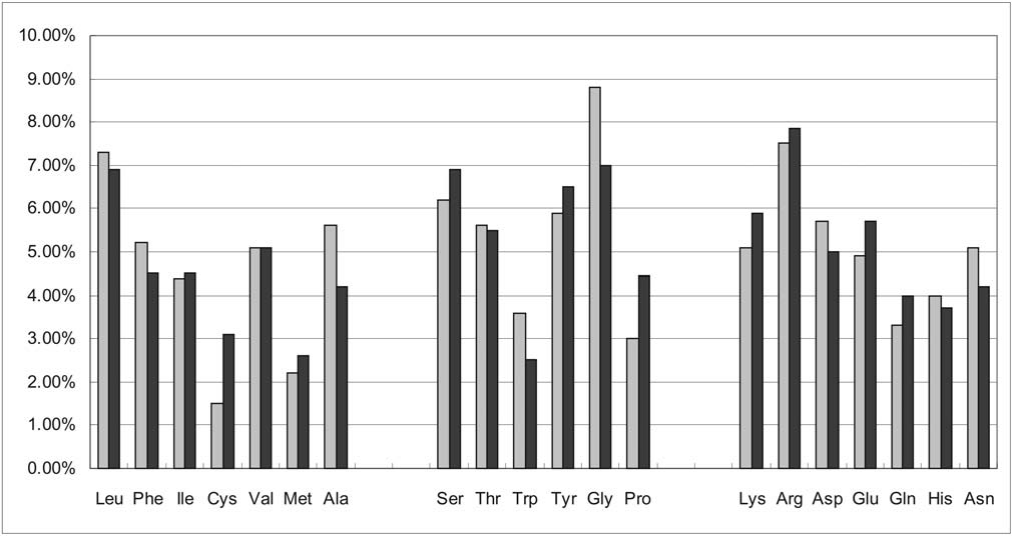
Percentage of occurrence of amino acids in the protein-organic compound binding pockets (gray bar) and in protein-protein interaction interfaces (black bar).

Since proteins and small organic compounds share similar chemical composition, we examined similarities and difference between AA composition of protein-organic compound binding pockets and protein-protein interaction interfaces, see Figure 8. The AA composition of protein-protein interaction interfaces (gray bars) is taken from [42], where it was calculated based on a non-redundant set of 170 protein-protein complexes. We note that except for Cys, which occurs twice as often in the protein-protein interaction interfaces when compared with the protein-organic compound binding pocket (3.1% vs. 1.5%), other AAs occur with similar frequency for both types of interactions. This similarity indicates that protein-ligand and protein-protein interactions could be determined by same types of forces and thus current protein-ligand docking techniques could be potentially adopted for protein-protein docking.

### Overlap and coverage of the interaction patterns

The 11 patterns that concern covalent bonds, coordination bonds and hydrogen bonds, see the bottom layer in Figure 1, appear in 2013 protein-organic compound complexes, 1138 protein-metal ion complexes, 1115 protein-anion complexes, and 53 protein-inorganic cluster complexes, which corresponds to (2013+1138+1115+53)/7759 = 55.7% of all protein-ligand complexes. Significant majority of the above complexes incorporates just one of the discussed patterns. More specifically, except for 81 protein-organic compound complexes and 546 protein-metal ion complexes that incorporate two or more interaction patterns, the remaining 4238 protein-ligand complexes include one interaction pattern.

### Prediction of binding sites of organic compounds based on interaction patterns

We show the utility of the discussed patterns in the context of the blind (without the knowledge of the ligand) prediction of binding sites. Since organic compounds are the largest group among the four considered types of ligands and since majority of the oral drugs are based on the organic compounds, we design and test a naïve method to predict the binding sites for the organic compounds that utilizes the knowledge of the four corresponding interaction patterns shown in Figure 1. The predictions are made using a dataset that consists of 901 proteins that was introduced in [43], in which the pairwise sequence identity is below 35%. Over 90% of these chains interact with only one organic compound, and the remaining chains interact with 2 or 3 compounds. Other types of ligands, e.g., metal ions, may bind to some of the chains, however, the binding sites of these ligands are not considered.

The prediction procedure follows a sequence of three steps:

1. Calculate a grid encompassing the protein structure using the Ligsite^CSC^ program [44]. The grid points are divided into those that are inside the protein structure, on the surface, and in the solvent [44]. We only use the solvent grid points.
2. Scan the solvent grid points that are within 5Å from the protein surface and count the interaction patterns that are within *R* distance from a given grid point. Only the atoms on the protein surface are considered. The interaction patterns for organic compounds include hydrogen bond (formed between NH- group of residue and oxygen atom of ligand) and covalent bonds. The actual counts of the hydrogen bonds and covalent bonds cannot be computed since this is a blind prediction. Instead, we count NH- group within the *R* radius to reflect potential hydrogen bonds. For the covalent bonds, we count sulfur atoms to reflect potential thioether and disulfide bonds.
3. Sort the grid points in the descending order based on the computed counts. The first prediction corresponds to the top scoring grid point. The subsequent predictions correspond to the points with the largest scores which are at least 5Å away from any accepted prediction.

The predictions are evaluated based on the distance between the predicted site and the actual position of the ligand, i.e., a prediction is assumed correct if the distance is smaller than a cut-off threshold value, which is varied between 1 and 10Å. For a given protein structure, 5 potential binding sites are predicted, which follows the procedure performed in relevant recent studies [44]. More specifically, if any of the 5 predicted binding sites is less than a certain distance (*D*) from any atom of the ligand, the prediction for this protein is assumed correct. This is motivated by the fact that the input dataset provides incomplete information, i.e., some actual binding sites could be missing which implies that some predictions that are far from the ligands included in the dataset could be potentially correct. The success rate is defined as the number of the correctly predicted proteins divided by size of the dataset.

**Figure 9:**
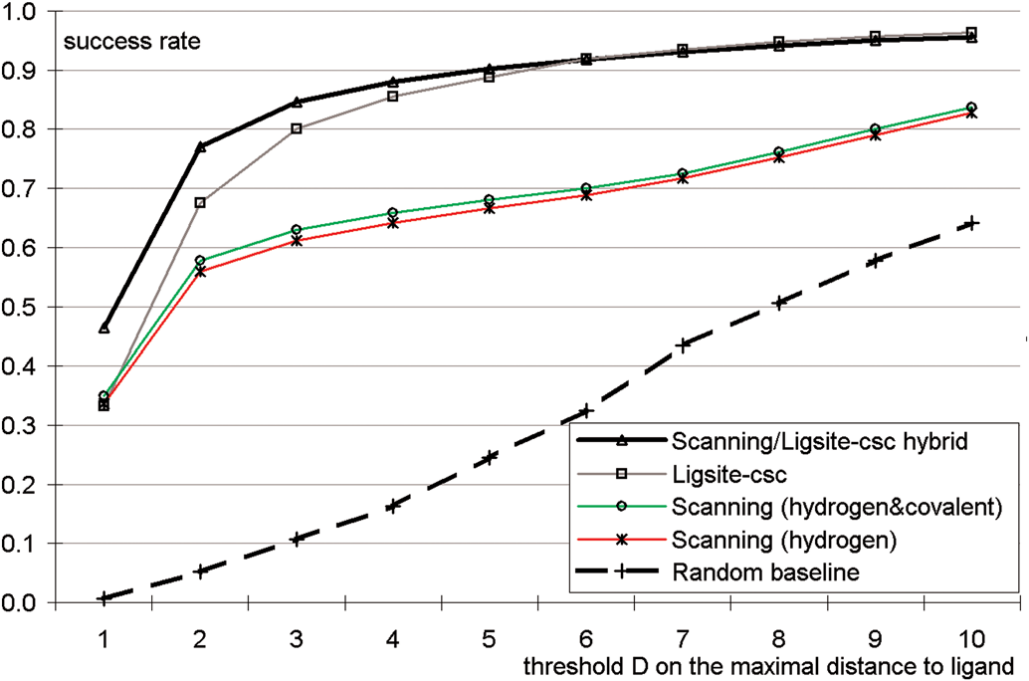
Performance of blind binding site predictors including the pattern-based method, Ligsite^CSC^, and a random baseline predictor. The *y* axis shows success rate, i.e., fraction of proteins with minimum distance between the top five predicted binding sites and any atom of a ligand in the native complex that is smaller or equal to the distance displayed on the *x* axis. The five plots concern the scanning method based solely on the hydrogen bond pattern (named “Scanning (hydrogen)”), the scanning method based on the four patterns concerning both hydrogen and covalent bonds (named “Scanning (hydrogen&covalent)”), the result of Ligsite^CSC^, the result of baseline method that randomly picks 5 solvent grid points that are within 5Å from the protein surface (named “Random baseline”), and the results that merge the top two predictions of Ligsite^CSC^ and the top three predictions of the scanning method that uses the four patterns (named “Scanning/Ligsite-csc hybrid”).

The pattern-based method is compared with Ligsite^CSC^ that identifies pockets on the protein surface based on a geometrical analysis [44], and which extends Ligsite method [45]. Ligsite^CSC^ is shown to perform comparably well or better when compared with several other binding site predictors such as Ligsite, CAST, PASS, and SURFNET [44]. We also implemented a baseline predictor by random selection of five solvent grid points that are less than 5Å from the protein surface. Two versions of our scanning-based approach are considered, one that uses both hydrogen and covalent bonds patterns, and the other that uses only the hydrogen bond pattern. The radius *R* is set to 8Å since for this value 1%–5% higher success rate is achieved when compared with other values between 5 and 10Å. Figure 9 compares the predictions. The naïve method based on the interaction patterns is inferior to Ligsite ^CSC^, i.e., our success rate is about 10% lower than that of Ligsite ^CSC^ for *D* = 2 to *D* = 10. This is not surprising since the four interaction patterns do not cover all protein-organic compound interactions as discussed above and since 29% of the organic compounds bind to protein only through van der Waals contacts. We observe that both methods are superior to the random predictions. We also observe that adding the patterns concerning covalent bonds improves the success rates by 1 to 2% across different *D* values, which shows that combining multiple patterns is helpful. This improvement is due to the inclusion of thiol of Cys, which potentially forms thioether and disulfide bonds.

**Figure 10:**
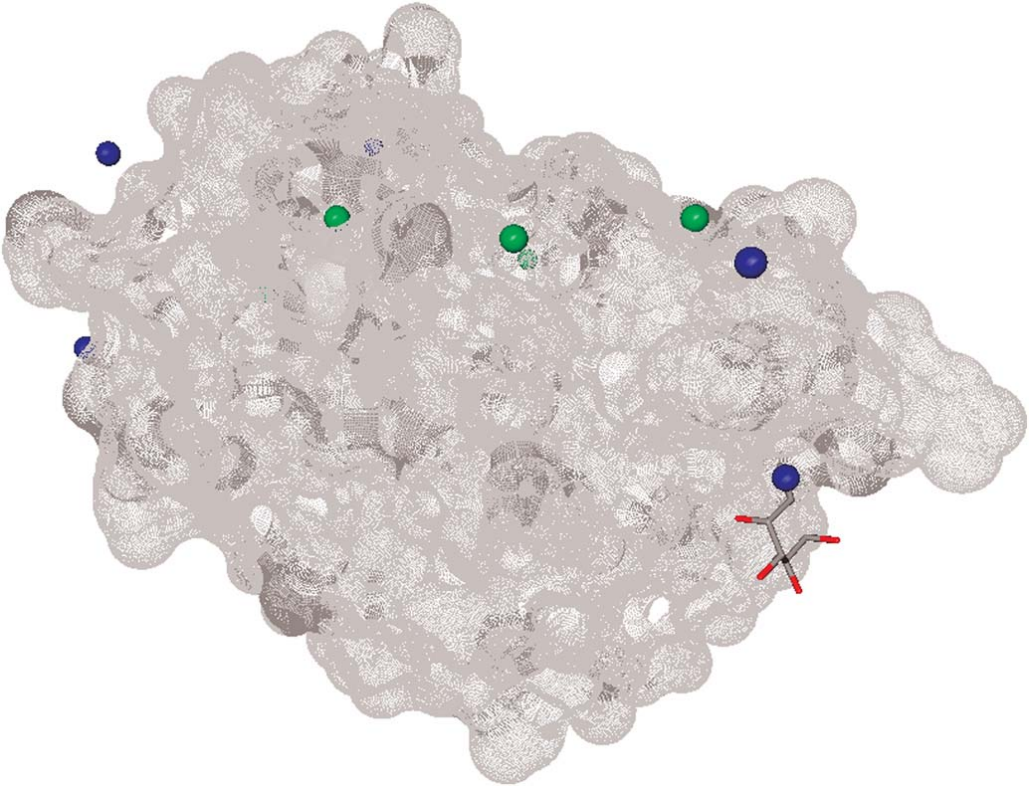
The structure of Anguilla anguilla agglutinin protein (PDB entry 1K12). The binding sites predicted by Ligsite ^CSC^ are colored in green and the binding sites predicted by the pattern-based method are colored in blue. The protein surface is rendered in gray and the ligand is in the stick form. The Ligsite^CSC^ predictions are over 10Å away from any atom of the ligand, while one of pattern-based predictions is 0.67Å away from one of the ligand's atoms. Only 4 predictions by Ligsite ^CSC^ and by the pattern-based method are visible; the remaining predictions are on the other side of the protein.

We observe that the prediction from the pattern-based method are complementary to the prediction from Ligsite ^CSC^, i.e., for some proteins the binding sites predicted by Ligsite ^CSC^ are relatively far away from the actual binding sites while our method provides correct predictions. For example, for the Anguilla anguilla agglutinin protein (PDB entry 1K12), the 5 prediction generated by Ligsite ^CSC^ are at least 11 Å away from the ligand, while one of our predictions is only 0.67Å from the compound, see Figure 10. This motivated a hybrid approach in which predictions from the two methods are combined by taking the top two predictions from Ligsite^CSC^ and the top three pattern-based predictions (on average the third best pattern-based prediction is better than third best prediction from Ligsite^CSC^). Figure 9 shows that the results based on the merged predictions are better than the results from individual methods, especially for low values of *D*. For instance, in the case of *D* = 1, both Ligsite ^CSC^ and pattern-based methods predict the binding sites that are within 1Å from the ligand for about 35% of the proteins, while the merged predictions are successful at 46% level. This result indicates that interaction patterns could be utilized to improve existing blind geometrical predictions of binding sites.

## Discussion

One of challenges in contemporary protein research is the discovery of generic rules and interaction patterns from the growing body of structurally characterized protein–ligand complexes. This study presents and investigates several frequently occurring interaction patterns for atomic-level protein-ligand interactions. The considered protein pocket-ligand complexes were grouped into four categories: protein-organic compound, protein-metal ion, protein-anion, and protein-inorganic cluster complexes. These groups cover 93.5% of all protein-ligand complexes from PDB and we show that they are governed by different types of interaction forces. The protein-organic compound complexes are governed by the hydrogen bonds, van der Waals contacts and covalent bond. The protein-metal ion complexes are based on the electrostatic force and coordination bonds while the protein-anion complexes are governed by the electrostatic force, hydrogen bonds and van der Waals contacts. Finally, the protein-inorganic cluster complexes are established mostly due to the coordination bonds.

We present several frequently occurring interaction patterns, defined in terms of prevalent interactions between specific atoms of specific residue in the protein's pocket and specific atoms of the ligand, for the abovementioned four groups and for the specific types of interaction forces. We quantify relative abundance of specific interaction types and discuss their characteristic features such as commonly interacting amino acid types. Total of 10 interaction patterns that occur in 56% of all considered complexes were found. For example, we show that 66.9% of the protein-organic compound complexes involve hydrogen bonds and that 65.8% of these hydrogen bonds are formed between the NH- group of the protein's residue and the oxygen atom of the organic compound. As a result, we believe that the geometric and electrostatic complementary, which are used for molecular recognition, should be supplemented by implementation of hydrogen bond(s) in the case of the protein-organic compound complexes. As another example, only four interaction patterns are sufficient to summarize significant majority, i.e., 73%, of normal covalent bond interactions between proteins and ligands; they include the covalent bond between the thiol of Cys residue and the carbon atom of the ligand (thioether bond), the thiol of Cys residue and the sulfur atom of the ligand (disulfide bond), and the nitrogen atom of Lys residue and the carbon atom of the ligand. We also show that the AAs serve as donors for significant majority of these hydrogen bonds. We observe that most of the inorganic anions interact with positively charged AAs including Arg, His, and Lys.

We show that the organic compounds form hydrogen bonds more frequently with hydrophilic AAs when compared with hydrophobic AAs, which is consistent with results obtained for protein-DNA interactions [5]. This suggests that the ability of AAs to establish hydrogen bonds could be an intrinsic characteristic of a given AA, which is independent of the ligand type. We also found that protein-organic compound binding pockets and protein-protein interaction interfaces share similar AA composition, which may imply that these interactions are determined by the same types of forces.

We also demonstrate a practical application of the abovementioned patterns in the context of blind prediction of binding sites for organic compounds. Our analysis reveals that a scanning method based on simple counts of the occurrence of the patterns provides predictions that complement existing methods that are based on the geometrical analysis of the protein surface.

To conclude, we show that for a given type (group) of ligands and a given type of the interaction force, majority of protein-ligand interactions are repetitive and could be summarized with several simple atomic-level patterns. These interaction patterns not only provide a comprehensive overview of protein-ligand interactions, but they also may have profound implications for development of molecular docking procedures and in building of binding site prediction methods.
